# Pyramiding of transcription factor, *PgHSF4*, and stress-responsive genes of *p68*, *Pg47*, and *PsAKR1* impart multiple abiotic stress tolerance in rice (*Oryza sativa L.*)

**DOI:** 10.3389/fpls.2023.1233248

**Published:** 2023-08-25

**Authors:** H. S. Sheela, Amaranatha R. Vennapusa, Kalpalatha Melmaiee, T. G. Prasad, Chandrashekar P. Reddy

**Affiliations:** ^1^ Department of Crop Physiology, University of Agricultural Sciences, Gandhi Krishi Vigyana Kendra (GKVK), Bengaluru, KA, India; ^2^ Department of Agriculture and Natural Resources, Delaware State University, Dover, DE, United States

**Keywords:** abiotic stress, multiple genes, transgenics, gene stacking, stress tolerance, trait, mechanism

## Abstract

Abiotic stresses such as drought, salinity, and heat stress significantly affect rice crop growth and production. Under uncertain climatic conditions, the concurrent multiple abiotic stresses at different stages of rice production became a major challenge for agriculture. Hence, improving rice’s multiple abiotic stress tolerance is essential to overcome unprecedented challenges under adverse environmental conditions. A significant challenge for rice breeding programs in improving abiotic stress tolerance involves multiple traits and their complexity. Multiple traits must be targeted to improve multiple stress tolerance in rice and uncover the mechanisms. With this hypothesis, in the present study gene stacking approach is used to integrate multiple traits involved in stress tolerance. The multigene transgenics co-expressing *Pennisetum glaucum 47* (*Pg47)*, *Pea 68* (*p68)*, *Pennisetum glaucum Heat Shock Factor 4(PgHSF4)*, and *Pseudomonas Aldo Keto Reductase 1* (*PsAKR1*) genes in the rice genotype (AC39020) were developed using the *in-planta* transformation method. The promising transgenic lines maintained higher yields under semi-irrigated aerobic cultivation (moisture stress). These 15 promising transgenic rice seedlings showed improved shoot and root growth traits under salinity, accelerating aging, temperature, and oxidative stress. They showed better physiological characteristics, such as chlorophyll content, membrane stability, and lower accumulation of reactive oxygen species, under multiple abiotic stresses than wild-type. Enhanced expression of transgenes and other stress-responsive downstream genes such as *HSP70, SOD, APX, SOS, PP2C*, and *P5CS* in transgenic lines suggest the possible molecular mechanism for imparting the abiotic stress tolerance. This study proved that multiple genes stacking as a novel strategy induce several mechanisms and responsible traits to overcome multiple abiotic stresses. This multigene combination can potentially improve tolerance to multiple abiotic stress conditions and pave the way for developing climate-resilient crops.

## Introduction

Climate change and its unpredictable consequences of environmental stress conditions have a significant negative impact on crop productivity, food security, and the global agricultural industry (Mickelbart et al., 2015; Varchney et al., 2018; Roychowdhury et al., 2020; Suprasanna, 2020). With the rapid changes in the climate and increasing population, there is a critical need to enhance agricultural production (Kumar et al., 2023). According to predictions, a 70% of increase in agricultural production is needed by 2050 to accomplish the needs of the expanding population and global challenge (Ortiz-Bobea et al., 2021; Swain et al., 2023). Major cereals such as rice, wheat, and maize are extremely vulnerable to various abiotic stresses, and the climate resilience of agriculture depends on the tolerance levels of the crop to multiple abiotic stresses that cause significant yield losses either individually or in combination (Rane et al., 2021; Roychowdhury et al., 2023). Among cereals, rice is an important food crop and is consumed by about three billion people all over the world (Roychowdhury et al., 2013). It is the staple food of the largest number of people in the world. In India, rice is cultivated round the year (in one or the other part of the country) in diverse ecologies spread over 44.6 million hectares with a production of 132 million tons and an average productivity of 2.96 tons per hectare (Samikshya, 2016). Changing climatic conditions allowing the rice to experience multiple abiotic stress at several stages of crop growth, thus aggravating the stress impact on crop establishment, development, and productivity by altering the physiological and biochemical functions (Roychowdhury et al., 2019; Manikanta et al., 2020). The simultaneous occurrence of multiple abiotic stresses effect is considerably higher in rice than individual stresses, which triggers yield loss and crop failure (Hasanuzzaman et al., 2015; Pandey et al., 2017). Several studies have reported the effect of abiotic stress on the phenology of rice crops, such as seed germination, seeding emergence, vegetative growth, flower and panicle growth, kernel filling, and yield (Jeyasri et al., 2021; Rui et al., 2022). In recent decades, several studies and rice crop improvement programs have focused on improving crop yield and quality under abiotic stress (Patra et al., 2020; Panda et al., 2021; Radha et al., 2023). However, less successful rates and little information are available on improving multiple stress tolerance in rice (Ali et al., 2022). Hence, crop improvement programs in rice need to be targeted to improve the multiple abiotic stress tolerance to overcome the adverse environmental conditions under climate change situations.

Plants handle abiotic stress through cellular level tolerance by maintaining metabolic homeostasis, cellular membrane stability, osmotic adjustments, management of oxidative stress, production of stress-responsive genes and proteins and secondary metabolites, and lipid reprogramming (Li et al., 2015; Nisarga et al., 2017; Kiranmai et al., 2018; Callwood et al., 2021; Mulozi et al., 2023). By altering the expression of many genes through complex signaling pathways, plants have developed adaptive mechanisms for abiotic stresses. Therefore, altering the genes that are involved in enhancing cellular level tolerance has been one of the approaches for developing crop plants with improved stress tolerance (Valliyodan and Nguyen, 2006). From this context, it is essential to improve the adaptation of rice to multiple abiotic stresses by targeting various traits to improve the cellular levels of tolerance, growth, and yield.

The existing evidence suggests that tolerant species have better mechanisms to survive stress, probably due to novel genes or functionally superior gene products compared to susceptible species (Ruthgrene et al., 2019). Pearl millet (*Pennisetum glaucum L.*) and Peanut (*Arachis hypogea L.*) have been reported for an inherent capacity to tolerate several abiotic stresses (Balasubramanium and Maheshwari, 1989; Geetha et al., 2009). Therefore, transferring genes or gene-regulating traits from tolerant species to susceptible species could improve crop stress tolerance, growth, and production. The events of central dogma possess proficient replication, transcription, and translation machinery. Several studies reported that, under stress conditions, transcription and translation are affected by altering the genes and gene products taking part in them (Fulda et al., 2010). Therefore, sustaining the regular transcription and translation mechanisms is essential under stressful conditions.

Several reports suggest that many RNA helicases and chaperones like HSPs (heat shock proteins) have improved protein turnover and stability under stress conditions (Krishna, 2004). The *Heat Shock Factors* (*HSF*) is a sequence-specific DNA binding protein that binds specifically to heat shock elements (HSE). *HSFs* have been identified as transcriptional regulators of heat shock genes, which encode heat shock proteins (HSPs) (Hasanuzzaman et al., 2013). HSP proteins play a crucial role in plant response to various abiotic stresses by maintaining protein homeostasis. Over-expression of *HSFA1b* provided tolerance to drought in Arabidopsis (Bechtold et al., 2013). High-level overexpression of the *HsfA2* gene confers increased tolerance to heat stress but also to salt/osmotic stress (Ogawa et al., 2007). The expression of *PgHSF4* in *P*. *glaucum* showed that the transcript level of *PgHSF4* increased the most in response to heat stress within 30 minutes of exposure (Reddy et al., 2009). A study by Karthik et al. (2019) reported that the *p68* RNA helicase is one of the prototypic members of the DEAD-box protein, which belongs to the most prominent family of RNA helicases with a molecular weight of 68 kDa protein. Tuteja and Tuteja (2004) reported that, since *p68* has helicase activity, it plays an important role in the unwinding of dsRNA in both 3′–5′ and 5′–3′ directions. In a previous study, it was found that helicase enhances biological processes such as transcription and translation. It is also involved in stabilizing protein synthesis and is interacting with DNA-protein complexes to alter gene expression (Andrisani et al., 2022). In another study, it was reported that *p68* provided tolerance to salinity stress by unwinding dsRNA formed under abiotic stress, and it facilitates the proper functioning of RNA metabolism (Ru et al., 2021). A recent study reported that *p68* over-expressed tobacco, rice, and soybean showed tolerance to salinity stress (Tuteja et al., 2014; Karthik et al., 2019). Based on these scientific strategies, the present study focuses on maintaining RNA and protein stability to improve the translational process and protein folding mechanism under stress conditions. *EIF4a is the* DEAD-box RNA-stimulated helicase. It assists translation initiation by unwinding 5’UTR secondary structures (Tuteja et al., 2008). The gene *Pg47* is another RNA helicase structurally and functionally similar to *EIF4a* RNA helicase (86% homology). This gene confirms a tolerance to salinity stress by removing the secondary structure formed in the t-RNA processing under stress conditions. *EIF4a* over-expressed Arabidopsis seedlings showed tolerance to salinity stress (Munoz and Castellano, 2012; Rao et al., 2017). *Aldo-keto reductases* (*AKRs*) are a family of NADH-dependent aldehyde and ketose reductase. It plays an important role in detoxifying the cytotoxic compounds of reactive carbonyl compounds such as malonaldehyde (MDA), methyl glyoxal (MG), Maillard products, and Amadori compounds), which are generated during abiotic stress conditions (Nareshkumar et al., 2020). Overexpression of *Pseudomonas AKR1* (*PsAKR1*) in rice and tobacco showed improved tolerance and seed viability under salt stress and seed aging stress (Nisarga et al., 2017; Ramu et al., 2017). The over-expression of *AKRs* from different plant and microbial species showed improved tolerance to multiple abiotic stresses such as salt, heat, drought, heavy metals, and oxidative stress, and these enzymes were reported as multitasking soldiers for diverse role in plant metabolic and stress defense (Turóczy et al., 2011; Sengupta et al., 2015; Nareshkumar et al., 2020).

Abiotic stress tolerance is complex and requires multiple traits to acquire plant tolerance. Hence, improving the various traits in crops may enhance tolerance to multiple abiotic stresses (Roychowdhury and Tah, 2011). Genetic engineering is a promising technology to transfer a gene from one species to another. Although alteration of a single gene has successfully improved certain plant characteristics, manipulation of composite interactive metabolic pathways and important quantitative attributes require the co-expression of multiple genes (Vemanna et al., 2012). In previous studies, the validation of individual transgenes (*PgHSF4*, *p68*, *Pg47*, and *PsAKR1*) in the model system suggests these transgenes expression could enhance crop plants stress tolerance (Patil, 2015; Nisarga et al., 2017; Salimath and Udayakumar, 2017). However, there are no reports of the combined expression of these genes together in response to abiotic stresses and multiple stress tolerance. With this hypothesis, we attempted to know the combined effect of all four genes through simultaneous expression in response to multiple abiotic stress and improved traits. In the present study, three different transgenes from hardy crop species, viz, *p68* from peanut and *Pg47* and *PgHSF4* (Heat Shock Transcription Factor 4) from *Pennisetum glaucum*, which are involved in maintaining RNA stability and translational processes and protein stability were co-expressed along with marker-free gene, *PsAKR*1 from *Pseudomonas* sp reported to involve in abiotic stress tolerance. The rice genotype AC39020 was reported to have superior characteristics of water mining, water use efficiency, and water conservation in response to drought (Vennapusa et al., 2015; Pushpa et al., 2023). The multigene-expressing rice lines were developed using the *Agrobacterium*-mediated *in-planta* transformation method, assessed the tolerance levels against multiple abiotic stresses, and characterized the physiological and molecular mechanisms responsible for tolerance and associated traits.

## Materials and methods

### Multiple gene vector construction and development of rice transgenics

The selected transgenes (*PgHSF4, p68* and *Pg47*) in the present study were expressed under different promoters and terminators, the *PgHSF4* gene was expressed under *2X35sCaMV35* (constitutive) promoter and *Poly A* terminator, and *p68* was expressed under *Ubiquitin* (constitutive) promoter and terminator and *Pg7* and *PsAKR*1 driven by the *RBCS* (constitutive) promoter and terminator. The single gene cassettes were cloned into entry vectors by restriction digestion. The *PgHSF4* gene cassette expressing *CaMV2x35S:HSF4* was cloned into a *pGATEL1-L4* entry vector using *Kpn I and Bam H I*. The vector *Ubi: p68* was digested with *Hind III* and *Eco RI* and cloned into the *pGATER4-R3* entry vector. Similarly, the *RBCS: Pg47* was digested with *Asc I* and *Pac I* and cloned into the *pGATEL3-L2* entry vector. The multigene cassette was developed by subjecting the entry vectors to the binary vector pi12GW containing *igrA* (*PsAKR1)* as a marker-free gene through LR clonase reaction (**Figure S1**) (Vemanna et al., 2012; Vennapusa et al., 2022). The developed multigene cassette was transferred into the *Agrobacterium*. The multiple genes co-expressing *PgHSF4*, *p68*, and *Pg47* cassette were transferred to rice cultivar AC39020 using *Agrobacterium-*mediated *in* ***-*** *planta* transformation to develop the transgenics (Vennapusa et al., 2022).

Physiological and molecular characterization of rice transgenic from T_0_ generation to T_3_ generation is depicted in **Figure S2**. In the T_3_ generation, to identify the promising transgenic lines, the plants were assessed for stress tolerance using salinity and accelerating aging stress treatment, and the identified lines were advanced to the T_4_ generation. In the T_4_ generation, selected transgenic lines of the T_3_ generation were screened again for stress tolerance by using salinity and accelerating aging stress to select the promising lines. Molecular characterization was carried out in selected transgenic lines, which showed higher tolerance to salinity, accelerated aging, and maintained higher productivity. In the T_5_ generation based on the molecular characterization and stress tolerance, 15 transgenic lines were identified, advanced to the next generations, and assessed to understand the physiological, and biochemical responses to the stress.

### Abiotic stress impositions and evaluation of rice transgenics for stress tolerance

#### Seedling level salinity induction stress

The selected 15 T_5_ transgenic lines were subjected to NaCl stress by using the salinity induction response technique (Jayaprakash et al., 1998). One set of germinated seedlings (10 seedlings for each line) was sequentially exposed to the gradual induction treatment of 50, 100, and 200 mM NaCl for 3 h each and subsequently exposed to a lethal concentration of 350 mM NaCl for 48 h, and another set of seedlings (10 seedlings for each line) was directly exposed to lethal stress (350 mM). Later the stress was alleviated after 72 h, and seedlings growth was measured and expressed in centimeters (cm).

#### Accelerating aging stress

An accelerated aging protocol was employed to screen the transgenic lines (10 seedlings for each line) (Dennis, 2005; Nisarga et al., 2017), in which seeds were subjected to high temperatures (45°C) and humidity (100%). The seeds were exposed to accelerated aging at 100% RH and 45°C temperature for 8 days, and seedling growth was measured (cm).

### Whole plant-level salinity stress imposition

In order to study the salinity stress response of transgenic plants, selected transgenic lines and wild-type plants were raised on sand media for 15 days. The 15-day-old seedlings were subjected to salinity stress using 300 mM NaCl containing half MS media for a week. After one week of stress imposition, one set of seedlings was used to measure the growth parameters, and another set of the seedling samples snap-frozen in liquid nitrogen was used for assessing the gene expression analysis.

### Evaluation of growth and productivity under two regimes of irrigated aerobic conditions (moisture stress)

The T_3_ rice transgenic lines (103) were raised in nursery beds and after 25 days old seedlings were transplanted in the containment field facility (net house facility). The spacing maintained was 25×25 cm and each row had 12 plants. When the plants were 80 days old, one of the plots was subjected to moisture stress at the reproductive stage (MR-II) and another plot was maintained as a control (MR-I). Stress was imposed based on the actual water requirement of the crop which is derived based on the evapotranspiration values of the crop (ETcrop). ETcrop is arrived at based on the reference evapotranspiration (ET_0_) and crop coefficient (Kc) of a particular time/period. ETcrop was calculated by using the formula as described below,


$${\text{ET}}_{0}\left(\text{mm}\right)=\text{PE x Kpan}$$

$$\text{ET crop}\left(\text{mm}\right)={\text{ET}}_{0}\text{ x Kc}$$

The following growth and yield parameters were recorded at the time of harvest: a) a number of productive tillers, b) a number of filled seeds, c) yield, and d) total dry matter (above-ground biomass). Shoot dry weight was recorded by drying plant samples separated from roots and seeds at 70°C in a drying oven for 72 h expressed in grams. Grain weight was recorded by air drying, expressed in grams (Qiuyuan et al., 2022). Total dry matter (TDM-above-ground part) was calculated by combining grain and shoot dry weight, expressed in grams. Drought susceptible index (DSI) was calculated by using the below-mentioned formula,


$$\text{DSI=}\frac{\left(\text{Yield under control}/\text{yield under stress}\right)}{\left(\text{Mean yield under control}/\text{Mean yield of stress}\right)}\times 100$$

### Evaluation of rice transgenic lines through biochemical analysis

#### Measurement of malondialdehyde (MDA) accumulation by thiobarbituric acid (TBA) reactive substances (TBARS) assay

The MDA content was determined according to Nareshkumar et al. (2020) with minor modifications. The samples were collected and homogenized in 2 ml of 0.1% (w/v) trichloroacetic acid (TCA). The homogenate was centrifuged at 10,000 rpm for 5 min. 0.5 ml of supernatant was added to the solution containing 4% TCA (w/v) and 0.5% TBA (w/v). The mixture was heated at 95°C for 15 min, then cool down to room temperature, and centrifuged at 10,000 rpm for 5 min. The clear solution’s absorbance was recorded at 532 nm and corrected for non-specific turbidity by subtracting the absorbance at 600 nm.

### Biochemical assessment for stress tolerance (NaCl stress) using excised leaf disc assay

The leaf samples of 65 days old plants were collected from NaCl-induced stress assay. The leaf discs were soaked in 300 mM NaCl for 2-3 days. After that, the leaf discs were washed twice with deionized water and used for the estimation of total chlorophyll content and membrane stability according to Arnon and Hoekstra methods, respectively (Arnon, 1949; Hoekstra et al., 2001).

### Heat stress

Two days old germinated seedlings of transgenic lines and wildtype (10 seedlings in each line) were incubated in agar media and provided heat stress (55°C) for 3 h and then incubated under room temperature for 8 days (Yufang et al., 2021). After 8 days of incubation, seedlings growth (cm) and protein content was assessed as described below and also were used for expression study.

### Total soluble protein quantification

The frozen leaf material from plants was ground in 100 mM Tris-HCl buffer (pH 7.8) containing 1 mM phenyl methyl sulfonyl fluoride (PMSF) and 5 mM benzamidine, and the solution was centrifuged at 12000 rpm for 10 min at 4°C. The supernatant was used for the quantification of protein. BSA standard curve was prepared (0.1-1.0 mg/ml) to estimate the protein concentration in the plant extract (Bradford, 1976).

### Methyl viologen (MV) stress

Two days old germinated seedlings were subjected to MV-induced oxidative stress. Seedlings (10 seedlings in each line) were incubated in agar media containing 8 µM solution of MV for 8 days. After 8 days of stress treatment, seedlings survival and seedlings growth (cm) was assessed (Nareshkumar et al., 2020).

### Nitro blue tetrazolium (NBT) staining assay

The extent of superoxide radicle production is determined with some modifications (Nareshkumar et al., 2015). One-week-old seedlings of both transgenic and wild-type were exposed to 300 mM NaCl stress for one day. Then the seedlings were immersed in a staining solution (2% NBT in 50 mM sodium phosphate buffer of pH 7.5) and incubated for 5 h till a blue color precipitate was observed from the clear staining solution. The extent of blue color staining formed was observed both in control and stressed seedlings.

### Diaminobenzidine (DAB) staining assay

The extent of hydrogen peroxide (H_2_O_2_) production was assessed with some modification (Nareshkumar et al., 2015). The seedlings of control and stress-exposed samples (300 mM NaCl for 1 day) were immersed in a staining solution (2.5 mM DAB in 10 mM citrate buffer of pH 3.8, filter sterilized and added 0.1% (V/V) tween 20) taken in a test tube. The seedlings were incubated for 5 h under high humidity conditions till a brown precipitate was observed from a clear staining solution. The extent of brown color formed was observed both in control and stressed seedlings.

### Evaluation of rice transgenic lines through molecular analysis

#### Extraction of plant genomic DNA

Genomic DNA was extracted from leaf tissue by using the CTAB method (Doyle and Doyle, 1990). PCR analysis was carried out using genomic DNA as a template for checking the integration of transgenes using both promoter and gene-specific primers (**Table S1**).

#### Isolation of total RNA

Total RNA was extracted from leaf tissues of both transgenic and wild-type using phenol–chloroform method with minor modifications (Datta et al., 1989; Vennapusa et al., 2020). The cDNA was synthesized by oligo (dT) primers using Moloney murine leukaemia virus reverse transcriptase (MMLV-RT; MBI Fermentas, Hanover, MD). *Actin* was used as an internal control for normalization (for the primer list, see **Table S1**). The PCR with SYBR dye and PCR conditions were 94°C for 5 min, 32-35 cycles of 94°C for 30s, 52–60°C for 30s, 72°C for 40s and a final extension of 72°C for 5 min. The expression profiles of *PgHSF4*, *p68* and *Pg47* genes were examined in the plants exposed to two different abiotic stress conditions (salinity and heat stress) with comparison to control conditions. Salinity stress was imposed by subjecting 15 days old seedlings to 300 mM NaCl treatment for 8 days. After 8 days of stress treatment, seedlings were kept 8 days of recovery and the leaf sample was collected on the 8^th^ day of stress recovery for expression studies. For heat stress, two days germinated seedlings were exposed to heat stress (55°C for 3h) and then incubated for 8 days. After completion of the incubation period, the seedlings were used for expression analysis of transgenes.

### Transgene DNA sequence analysis

PCR amplification of the *PsAKR1 (igrA)* gene was performed using genomic DNA. The PCR gene product amplified from genomic DNA isolated from transgenic leaf samples was resolved on 0.8 percent agarose gel. The fragments of interest were eluted and purified from the gel by using Gen Elute Gel Extraction Kit (Sigma Aldrich, USA). The purified PCR gene products were sequenced, and the obtained sequences were analyzed by CLASTALW and confirmed the integrated transgene sequences.

### In-silico analysis of *p68* and *Pg47* gene-associated protein interactions and validation of gene expressions

Protein-protein interaction study was carried out using an online STRING 10 bio-analytical tool (http://string-db.org/).

### Statistical analysis

ANOVA (Analysis of variance) was used to analyze the significant difference between control and stress conditions (Fisher, 1925). The level of significance used in the F-test and t-test was P = 0.05.

## Results

### Generation of transgenic rice plants

The multigene construct (*PgHSF4*, *p68*, and *Pg47*) was developed in the background of binary vector *pi12GW* which contains a selectable marker-free gene *PsAKR1*, which is resistant to glyphosate and other abiotic stresses (Vemanna et al., 2017; Vennapusa et al., 2022). The overall construct details are provided in **Figure S1**. The transgenic rice plants co-expressing *PgHSF4*, *p68*, and *Pg47* were developed by *Agrobacterium*-mediated *in* ***-*** *planta* transformation. The putative T_0_ rice transformants were selected based on the selection screening with glyphosate and the putative transformants were advanced to the next generations. Details of the advancement of transformants to T_1_ and T_2_ generation and screening selection protocol are depicted in **Figure S2**. Further, functional characterizations of transgenics in T_3_ generation were carried out against abiotic stress to identify the promising lines with superior stress tolerance.

### Advancement of multigene rice transgenics and identify the promising lines based on responses to various abiotic stresses

**Figure 1:**
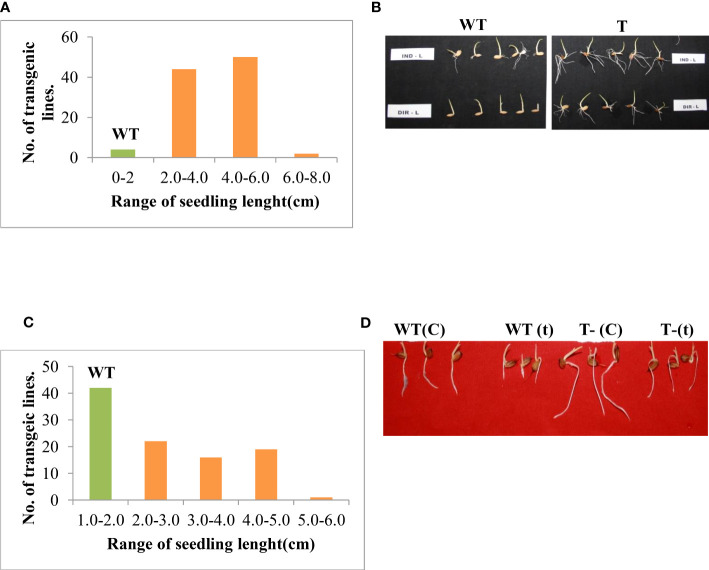
Screening of T_3_ putative transformants against different abiotic stresses. **(A)** Frequency distribution of rice transgenics based on root and shoot length for salinity stress response. **(B)** Photograph showing the salinity stress response of transgenic rice line (T-50). **(C)** Frequency distribution of rice transgenics based on root and shoot length for accelerated aging stress response. **(D)** Growth response of transgenic lines under accelerated aging stress. WT-wildtype, T-transgenic, C-Control, t- treated.

The level of stress tolerance in the T_3_ rice transgenics was investigated using a salinity induction response stress assay. The transgenic lines maintained high recovery growth compared to wildtype. However, there was a considerable variation in the response of transgenic lines to the salinity stress. The transgenic lines were categorized into two groups, the lines that maintained seedling growth up to 2-4 cm were grouped into moderately tolerant, and the lines that maintained seedling growth up to 4-6 cm were grouped as highly tolerant lines based on growth recovery after the stress imposition (**Figures 1A, B**). To assess the seedling vigor upon stress, the T_3_ transgenic lines were evaluated using another technique i.e, Accelerating aging. The transgenic lines maintained high seedling vigor and growth compared to wild-type upon accelerated aging. In response to accelerating aging, transgenic lines showed considerable variation, the transgenic lines, which maintained growth from 2-3 cm, were grouped into moderately tolerant (2-3 cm) and the seedlings that maintained growth from 3-5 cm were categorized as highly tolerant lines (3-5 cm) (**Figures 1C, D**). The highly tolerant lines based on both salinity stress and accelerating aging were selected and advanced to the next generation for further characterization.

### Evaluation of growth and productivity of T_3_ transgenic lines under two regimes of irrigated aerobic conditions (regime1-control, regime2- moisture deficit stress)

One hundred three lines of transgenic lines were raised in contained field conditions, and one set was subjected to moisture deficit stress at the time of flowering and compared with the wildtype lines. Stability in grain yield was estimated by the drought susceptibility index (DSI). DSI is one technique to detect drought resistance differences and quantify yield loss under moisture-stress situations. Besides low DSI value, another important criterion to identify the superior events is absolute productivity under stress. Therefore in the present study, both absolute productivity and DSI value were used to determine the promising transgenic lines which showed higher productivity under stress conditions. The study showed a strong negative correlation between DSI value and productivity under stress conditions (**Figure S3**). This signifies that the transgenic lines showed lower DSI values showed higher productivity under stress.

Based on the above-mentioned physiological stress assay and DSI value, 43 transgenic lines were selected and advanced to T_4_ generation. In the T_4_ generation, selected transgenic lines were stringently screened once again using salinity induction stress, accelerating aging stress, and evaluation under contained field conditions (**Tables S2**–**S4**). Based on these experiments (both physiological and molecular analysis), 15 superior tolerant transgenic lines were identified and advanced to the T_5_ generation. Further, the integration of all three genes in the tolerant lines was confirmed using PCR analysis.

### Promising transgenic lines response to various abiotic stresses and molecular characterization

#### Response of T_5_ rice transgenic to salinity stress (350 mM NaCl) at the seedling level

**Figure 2:**
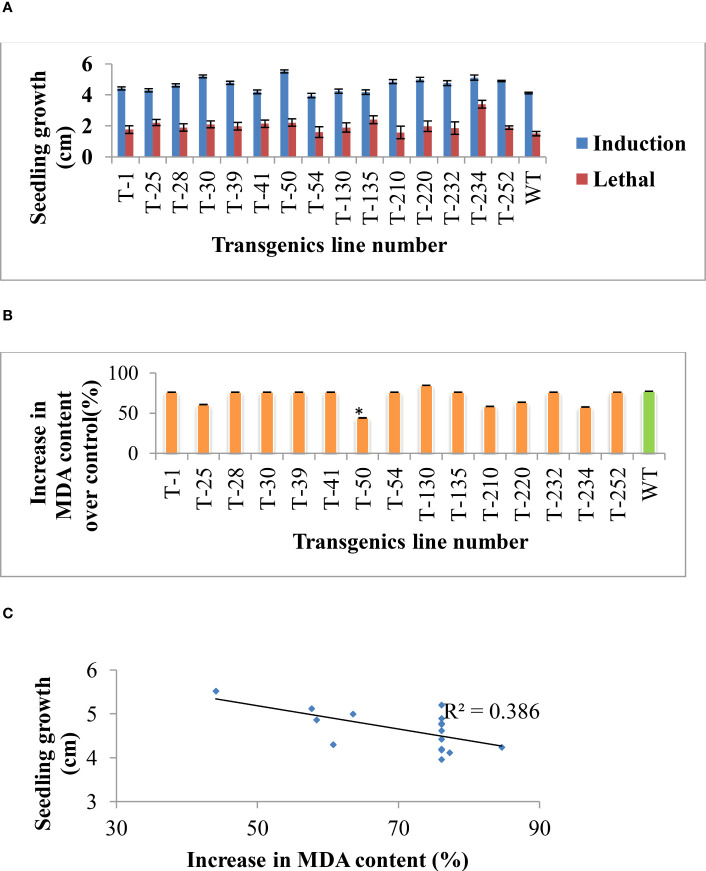
Rice transgenic seedlings’ growth response under NaCl-induced stress. **(A)** Germinated seeds were placed on 50, 100, and 200 mM NaCl (Induction treatment) for three h and transferred into the 350 mM NaCl (lethal concentration). After three days of stress treatment, seedlings were placed for recovery and shoot, and root lengths were recorded. **(B)** MDA estimation from control and NaCl-stressed samples (induction treatment sample). **(C)** Correlation between the seedling’s growth and percent increase in MDA content over control. Bars indicate standard error of replication, an asterisk (*) indicates the significance at P=0.05.

As evaluated in the earlier generation, selected T_5_ generation transgenics were assessed to identify superior transgenic lines. Transgenic lines’ recovery growth was significantly higher than wild-type (**Figure 2A**). The increasing effect of salinity stress causes oxidative stress by increasing the production of reactive oxygen species. Apart from reactive oxygen radicle, MDA is one of the final products of the oxidation of polyunsaturated fatty acids. Therefore, MDA content was measured for both control and stressed samples to know the effect of salinity stress caused membrane damage. Out of 15 transgenic lines, five transgenic lines (T-25, T-50, T-210, T-220 and T-234) showed significantly lesser MDA content than wild-type (**Figure 2B**), and the correlation curve between the seedling growth and MDA content shows a negative correlation upon salinity induction treatment (**Figure 2C**). The result revealed that transgenic lines that maintained higher seedling growth (Root and shoot length) upon salinity stress showed a lesser percent increase in MDA content, indicating less accumulation of MDA in transgenics due to effective management of oxidative damage caused by the salinity stress compared to wild-type.

#### Response of T_5_ rice transgenic to salinity stress (350 mM NaCl) in leaf disc assays at the whole plant level

**Figure 3:**
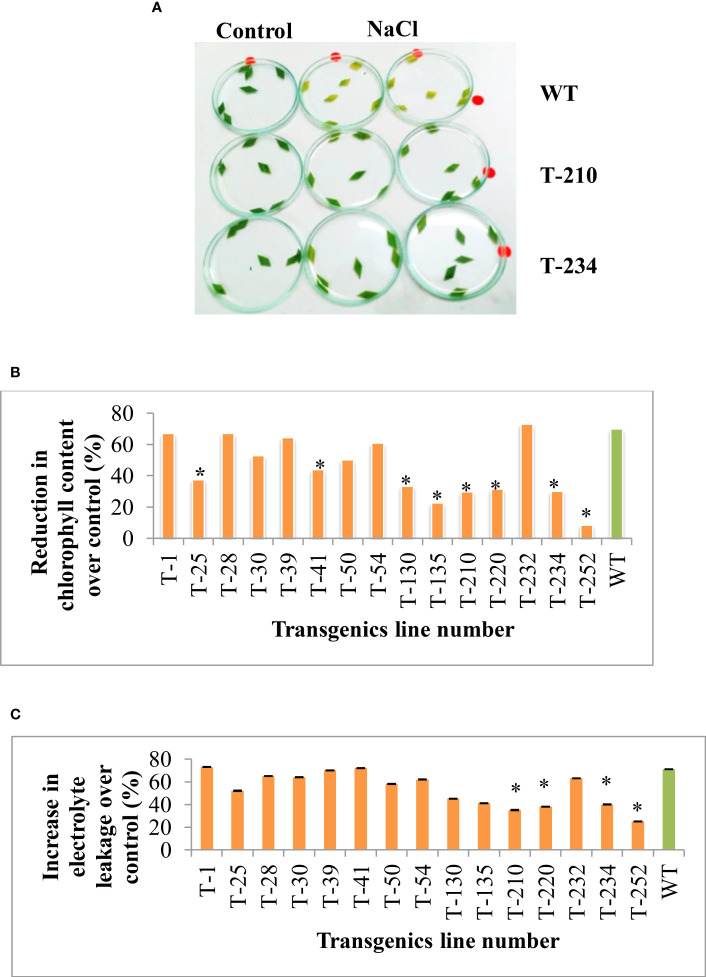
Response of rice transgenics to NaCl stress upon excised leaf disc assays. Leaf discs from transgenic and wild-type plants were placed on 350 mM NaCl,. **(A)** Photograph was taken 72 h after exposure to NaCl treatment. **(B)** Percent reduction in chlorophyll content **(C)** Percent increase in electrolyte leakage. Bars indicate a standard error of three replications, and an asterisk (*) indicates the significance at P=0.05, WT-wild-type, 1 to 252- different transgenic lines.

The cellular level tolerance of the T_5_ rice transgenic plants was assessed using the leaf disc assay. The transgenic leaf discs showed less visible yellow color symptoms compared to wild-type plants (**Figure 3A**). Further, the extent of NaCl-induced salinity stress damage was assessed by estimating the total chlorophyll content and electrolyte leakage. Salinity stress significantly affected the total chlorophyll content. However, the amount of reduction was less in transgenics than in wild-type (**Figure 3B**), and this NaCl-induced salinity stress also causes an alteration in the cell membrane stability by increasing the leakage of electrolytes. Many transgenic lines showed a significantly lesser increase in electrolyte leakage over the control compared to the wild-type (**Figure 3C**). The results indicate that NaCl stress-caused impact was less on rice transgenic chlorophyll content and cell membrane stability compared to wild-type.

### Growth and yield analysis of selected rice transgenics and wildtype plants

T_5_ transgenic lines and wild type were raised in contained field conditions, the growth and yield parameters were assessed, and spikelet fertility was calculated by counting total number of filled and unfilled seeds. The wild-type and some transgenic lines were severely affected under moisture stress conditions and there is a marked reduction in spikelet fertility, resulting in substantially reduced productivity. However, the percent fertility is relatively high in transgenics compared to wild-type (**Table 1**). Consequently, an increase in total dry matter is observed in transgenic lines. Based on the response to abiotic stresses at seedling and whole plant levels, five transgenic lines (25, 50, 210, 220, and 234) were selected as superior lines for further studies to understand the mechanisms of abiotic stress tolerance.

**Table 1:** Growth and productivity of T_5_ rice transgenics grown under contained field conditions.

Line numbers	Grain yield (g/plant)	Number of productive tillers	TDM (g/plant)	Spikelet fertility (%)
T-1	55.24*	5.67	109.20*	92.64*
T-25	46.84	6.00	92.07	90.54*
T-28	45.54	5.89	92.77	92.54*
T-30	56.34*	5.56	108.60*	92.23*
T-39	51.56	5.78	102.70	90.62*
T-41	45.78	5.33	93.11	97.31*
T-50	44.78	5.44	97.33	92.24*
T-54	45.60	6.56	104.00*	92.93*
T-89	46.00	5.56	92.00	90.64*
T-108	47.43	4.44	95.21	91.74*
T-130	53.31	5.67	107.50*	88.57
T-132	50.89	5.33	99.67	92.72*
T-135	51.54	5.22	96.43	90.80*
T-139	50.10	6.00	104.30*	94.17*
T-210	46.39	5.67	90.94	80.41
T-220	49.33	5.44	98.56	85.70
T-232	49.28	5.00	103.90*	88.21*
T-234	45.20	4.78	98.09	91.28*
T-252	49.22	6.22	93.22	97.07*
**T(Average)**	**48.97**	**5.56**	**98.93**	**91.18**
**T(Range)**	**44.7-56.3**	**4.4-6.5**	**90.9-109.2**	**80.4-97.3**
**WT(Average)**	**38.93**	**4.36**	**78.80**	**81.00**
**WT(Range)**	**34.4-45.6**	**3.7-4.8**	**70.4-87.6**	**78.3-83.9**
**CD (0.05)**	**15.25**	**2.75**	**24.71**	**5.5**

Bold value = indicates average and range of transgenic and wild type.*= indicates significance at P=0.05.

### Molecular characterization of T_5_ rice transgenics

Genomic DNA was isolated from both transgenic and wild-type plants. PCR amplification of genomic DNA was carried out by using *Actin* gene primers as an internal control and transgenes using gene-specific primers. The integration of the marker gene, *PsAKR1* in transgenic plants was confirmed using gene-specific primers with the amplification of 593 bp product, *PgHSF4* with the presence of 359 bp amplicon, *Pg47*, the 560 bp amplicon and *p68* with the application of 400 bp product (**Figure S4**). Further, to reconfirm the stable gene integration of transgene *PsAKR1*, the PCR-amplified product from genomic DNA was sequenced and analyzed by CLASTALW to check the homology between the original cloned sequence and the PCR product sequence. The PCR product of *PsAKR1*gene amplified from the transgenic lines showed 93% sequence homology with the original cloned sequence (**Figure S5**). This result indicates the integration of gene construct in the transgenics lines.

### Understanding the physiological, biochemical, and molecular basis for the improved multiple stress tolerance in selected promising transgenic lines under different abiotic stresses

**Figure 4:**
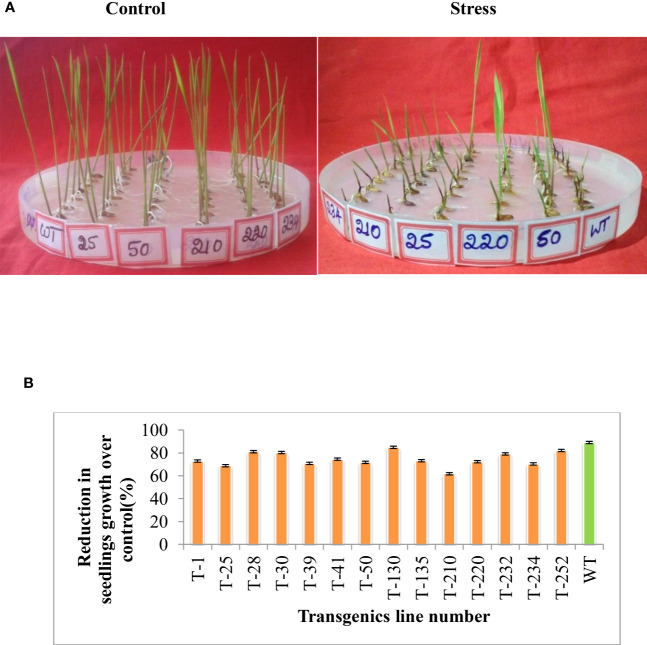
Multigene rice transgenics response to methyl induced viologen-induced oxidative stress. Two days germinated seedlings were placed on 0.6 µm methyl viologen for eight days. **(A)** shows the rice transgenic seedlings growth in response to methyl viologen stress. **(B)** Percent reduction in seedling growth of transgenics compared to wild type. WT- wild-type, 1 to 252-Transgenic lines. Error bars indicate the standard error of three replication.

**Figure 5:**
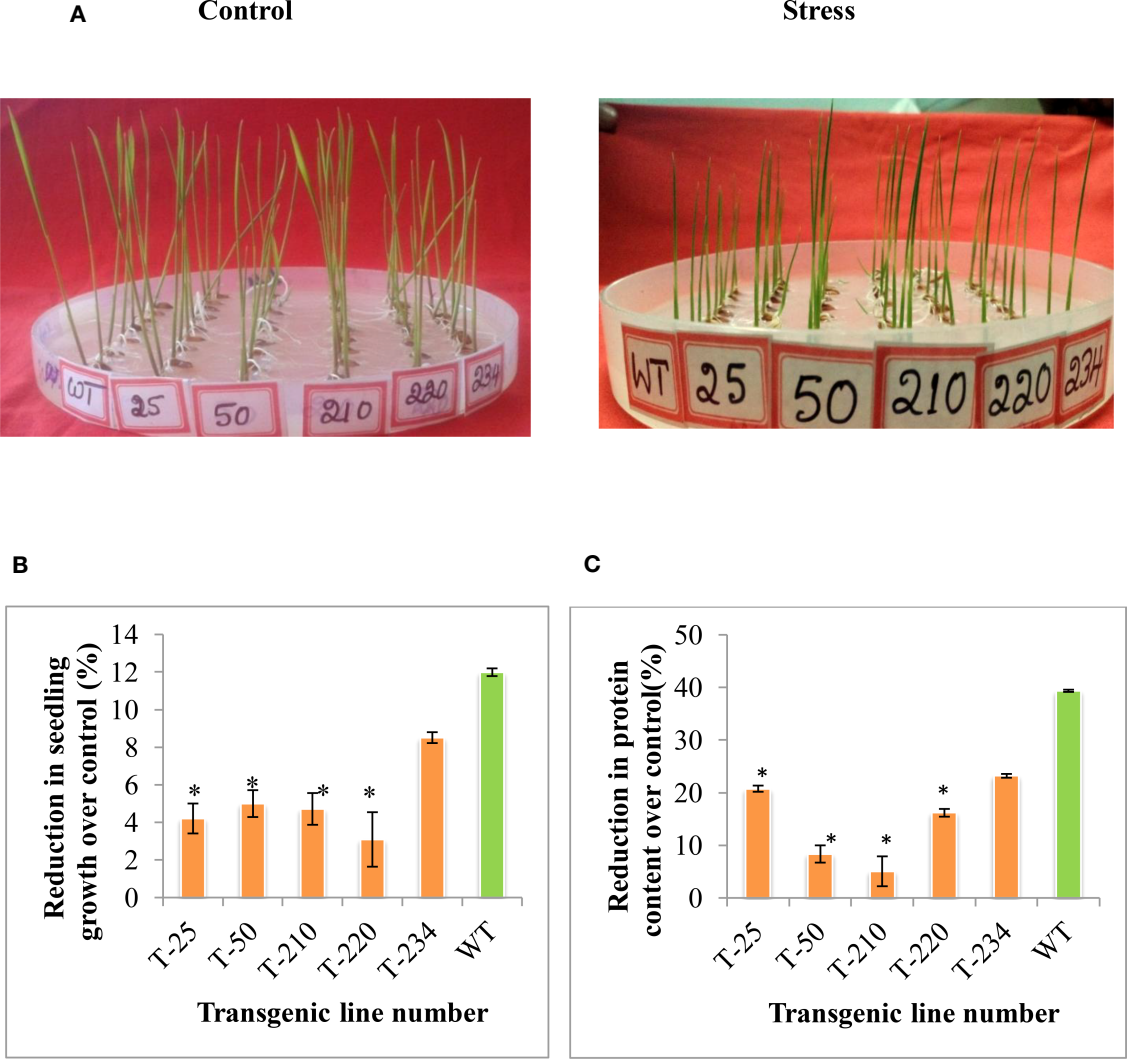
Response of multigene rice transgenics to heat stress. Two days-old germinated seedlings were subjected to heat stress for three h and then kept at room temperature for eight days. **(A)** Photograph showing the rice transgenics seedling growth response upon exposure to the heat stress. **(B)** Percent reduction in seedling growth over control, **(C)** Percent reduction in protein content over control. Error bars indicate the standard error of three replication, and the asterisk (*) indicates the significance at P=0.05.

To understand the stress tolerance levels and the basis for the tolerance, the selected five T_5_ transgenic lines were subjected to different abiotic stresses, such as heat stress and methyl viologen (MV**)**. To assess the effect of MV-induced oxidative stress in these selected five transgenic lines, two-day-old germinated seedlings were subjected to MV (8 µm) stress. The extent of MV-induced stress was measured in terms of seedling growth and stress conditions. The transgenic lines maintained better seedling growth compared to wild-type upon MV stress (**Figures 4A, B**). In response to heat stress (55°C for 3 h), the transgenic lines showed better seedling growth compared to wild-type (**Figures 5A, B**), and the reduction in total protein content was less in transgenic compared to wild-type (**Figure 5C**).

### Quantification of reactive oxygen species using histochemical assays

**Figure 6:**
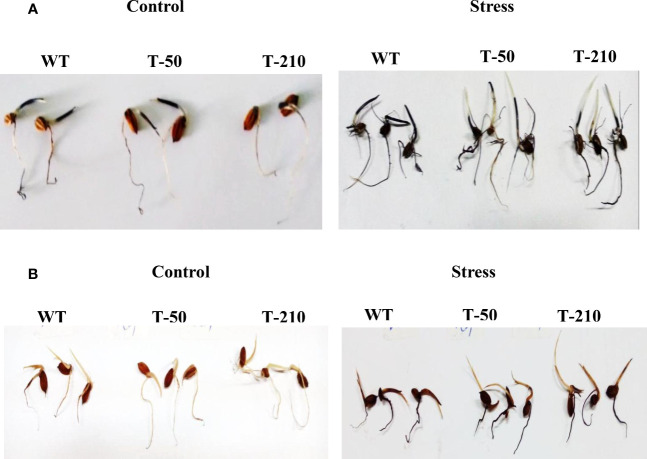
Quantification of reactive oxygen radicle using the histochemical assay. **(A)** One-week-old seedlings of wildtype and transgenic was incubated in 300 mM NaCl for 24 hours, seedlings were stained with NBT solution for quantification of superoxide radicles and **(B)** DAB solution staining for H_2_O_2_ quantification. WT-Wild-type, T50 and T210 – Transgenic lines.

The quantification of O^2-^ and H_2_O_2_ using NBT and DAB staining was assessed upon NaCl stress. One-week-old, both transgenic and wild-type seedlings of control and NaCl (300 mM) treatment were immersed in NBT and DAB solution. Under stress conditions, the wild type showed a higher accumulation of purple formazon compound than the transgenics. However, no significant differences were observed under the control conditions (**Figure 6A**). Similarly, when stressed seedlings were incubated in DAB staining solution, transgenic seedlings accumulated less H_2_O_2_, while deeper staining was observed in wild-type seedlings. However, under the control condition, there was no significant difference in the accumulation of dark brown colored compounds between wild-type and transgenics (**Figure 6B**).

### Gene expression profiles in response to salinity and heat stresses

**Figure 7:**
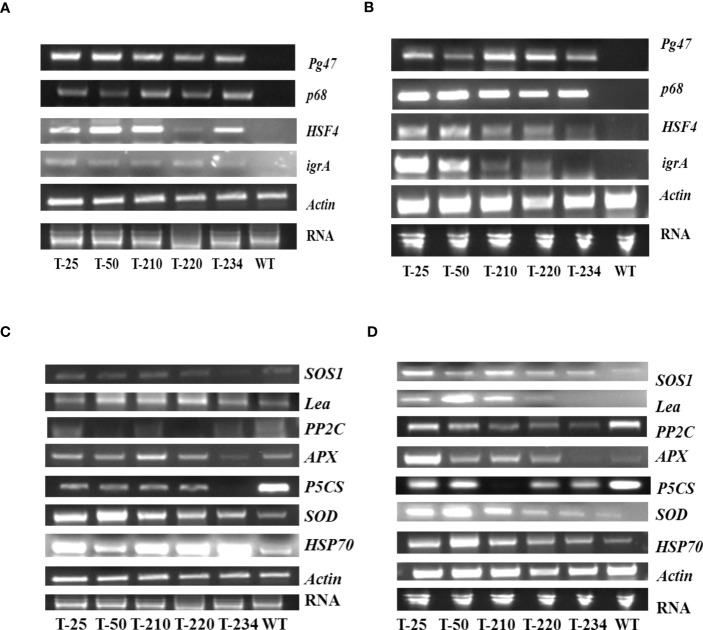
Expression analyses of multigene-expressing rice transgenic plants. **(A)** Semi-quantitative expression of transgenes under heat stress, **(B)** Gene expression profiles of transgenes under salinity stress, **(C)** Semi-quantitative expression analysis of downstream genes in multigene-expressing transgenics under heat stress conditions, and **(D)** Semi-quantitative expression analysis of downstream genes in multigene-expressing transgenics under salinity stress conditions. The cDNA was amplified with gene-specific primers, and actin was used as a housekeeping gene. WT – Wild-type, T-25 to T-234 – Transgenic lines.

The expression of transgenes and downstream regulated genes in response to abiotic stress was investigated. Under both NaCl and heat stress, transgenics had maintained high transcript levels of all three genes, including *PsAKR1* (**Figures 7A, B**). Among the transgenes, *HSF4* transcript levels were found to be higher compared to the other two transgenes in heat-induced stress (**Figure 7A**). While in salinity stress, the *p68* gene transcript level was higher compared to the other two genes (**Figure 7B**). However, expression of all these transgenes was not observed in wild-type. To understand the functional role of transgenes in abiotic stress tolerance, the expression profiles of the downstream/stress-responsive genes have been carried out. Under NaCl stress-showed a higher accumulation of *HSP70, SOD, APX, SOS*, and *LEA3* transcripts in the transgenics compared to wild-type (**Figure 7D**). Reduced expression levels of *PP2C* and *P5CS* genes were found in the transgenics under both salinity and heat stress (**Figures 7C, D**).

### Transgenes-protein interactions and validation of their internal expression in transgenic plants

Insilco analysis of *p68* and *Pg47* genes using protein-protein interaction studies was carried out to understand the unrevealing mechanism of the plants. Results showed that *Pg47* and *p68* are interacting with other proteins such as Zinc finger protein, *eIF4E, eIF4G, eIF4F* (Eukaryotic translation initiation factors), *MA3* domain-containing proteins, and *OsRNA helicases* (**Figure S6**). To study the gene expression of these interacting proteins, semi-quantitative RT-PCR analysis was carried out in the transgenic lines along with wild type. The gene expression of all interacting proteins was higher in transgenic lines compared to wild-type (**Figure S7**). The result indicated that all these interacting proteins might be attributed to abiotic stress tolerance induced by the transgenes of *p68* and *Pg47*.

**Figure 8:**
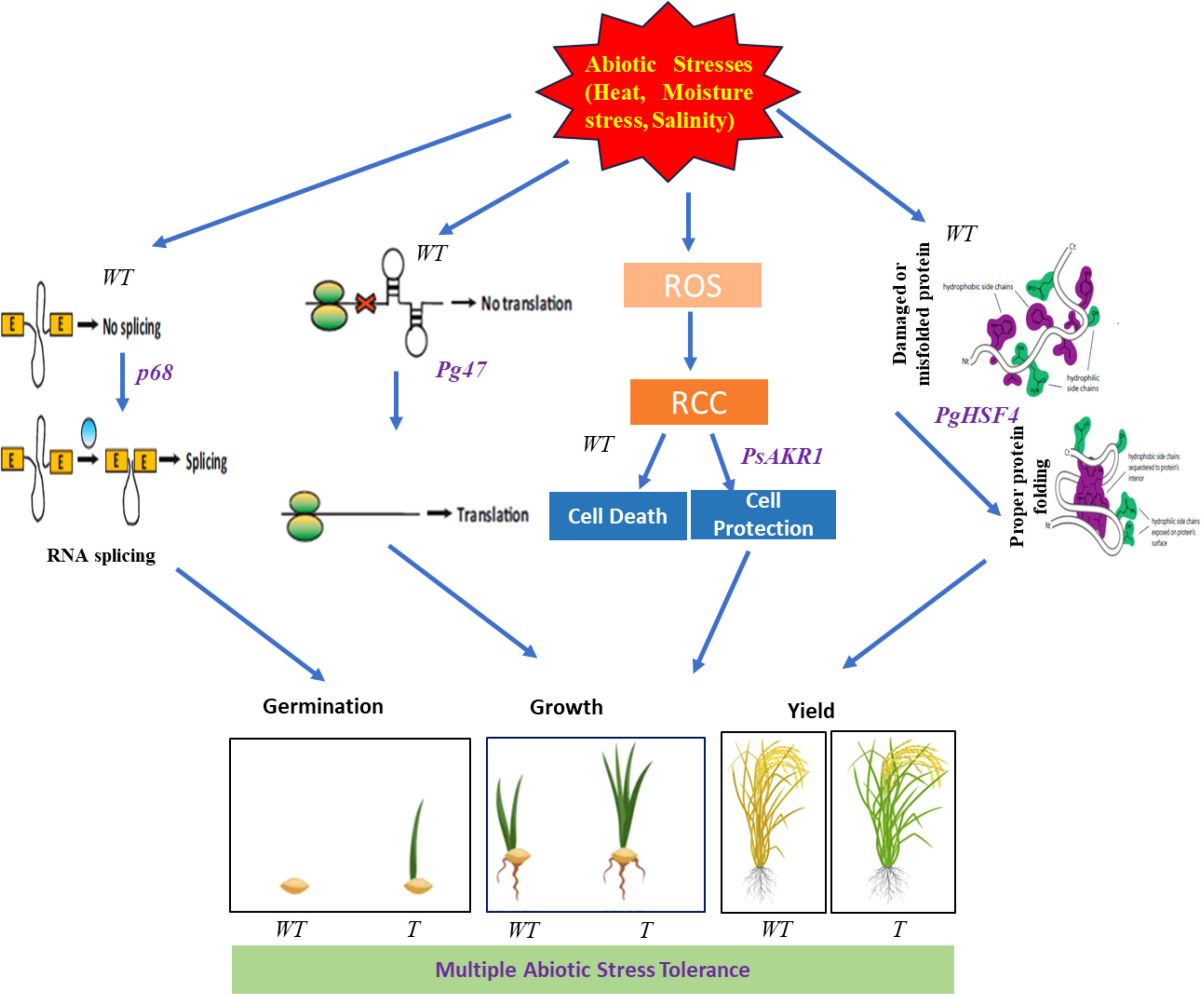
Overview of the stress tolerance mechanisms in multigene-expressing plants. The proposed model of multiple abiotic stress tolerance in multigene rice transgenics expressing *PgHSF4, p68, and Pg47 genes along with PsAKR1*. WT-Wild-type, T-Transgenics, ROS- Reactive oxygen species, RCC- Reactive carbonyl compounds. Pictures were adopted from Google and Vector Stocks.

All these results demonstrated that, transgenic lines showed improved tolerance to different abiotic stresses. Overview of the stress tolerance mechanisms in multigene-expressing plants is depicted in the **Figure 8**.

## Discussion

Plant abiotic stress tolerance is a genetically complex and multigenic trait, hence transferring single gene or trait is not sufficient to achieve improved abiotic stress tolerance in the crops. Therefore, the gene stacking approach can consider a potential approach to engineering abiotic stress-tolerant crops by introducing multiple traits and manipulating several-stress tolerant and regulatory mechanisms. Pyramiding more than one transgene may offer more effective tolerance than one gene in transgenic crops (Vemanna et al., 2015; Venkatesh et al., 2019). Several earlier studies reported that the pyramiding of multiple genes in transgenic crops exhibited tolerance against different abiotic stress. Simultaneous expression of regulatory genes such as *Heat Shock Transcription Factor* (*HSF4)*, *Pea DNA helicase* (*PDH45)*, and Alfalfa zinc finger 1 (*Alfin 1*) in peanuts showed improved tolerance to drought stress (Vemanna et al., 2015). Co-expression of abiotic stress-responsive transcription Factors such as *AtDREB2A, AtHB7*, and *AtABF3* delivered improved salinity and drought tolerance in peanuts (Pruthvi et al., 2014). Venkatesh et al., 2022 reported the pyramiding of multiple genes belonging to three different transcription factor families, *MuMYB96*, *MuWRKY3*, and *MuNAC4*, from a hardy crop horse gram (*Macrotyloma uniflorum*) showed improved drought stress tolerance by acquiring the water conservation, water mining, and cellular level tolerance traits in groundnut, and maintained sustained yield and physiological functions. Co-expression of *eIF4A1* with *NHX1* (Na +/H + antiporter), enhances tolerance to drought in sweet potato by improving ROS scavenging activity and by maintaining membrane integrity (Zhang et al., 2019). Likewise, in the present study, genes stacking (*HSF4, p68* and *Pg47*) helped rice plants overcome several abiotic stresses such as salinity, moisture stress, heat and accelerating aging.

Tuteja et al. (2014) reported that over-expression of *p68* in transgenic tobacco plants showed enhanced tolerances to salinity stress by scavenging of reactive oxygen species through antioxidant machinery. MDA is one of the reactive carbonyl compounds formed by the oxidation of polyunsaturated fatty acids under stress conditions. It is recently considered one of the indicators of the extent of stress experienced by plants (Nisarga et al., 2017). In our study, transgenic rice lines subjected to gradual salinity stress showed lower accumulation of MDA content compared to wild-type (**Figures 2A, B**). It indicates transgenics are effectively involved in the detoxification of cytotoxic compounds (*HSF4*, *p68*, *Pg47* and *PsAKR1*). Previous studies also reported that the over-expression of *PsAKR1* reduced the cytotoxicity caused by the salinity stress in tobacco transgenics (Vemanna et al., 2017).

Chlorophyll content and cell membrane stability are other important physiological traits, which are significantly affected by abiotic stress (Muhammad et al., 2019). Reduction in chlorophyll content and increase in electrolyte leakage are less in transgenics compared to wild-type (**Figures 3B, C**). The study’s result reveals that salinity tolerance in transgenic could be due to the over-expression of *p68* and *PsAKR1* genes. Therefore in the gene expression study, the transcript level of *p68* is more under salinity stress compared to *HSF4* and *Pg47* (**Figure 7B**), and transgenic also showed an enhanced expression of scavenging enzymes (SOD, APX) compared to wildtype (**Figure 7D**). Therefore the obtained result predicted that transgenic showed improved tolerance by decreasing ROS production (**Figures 6A, B**) through antioxidant machinery.

Seed aging is a combined function of time, temperature, and moisture (Ellis and Roberts, 1981). This seed aging subsequently causes seed deterioration by affecting the viability and vigor of seeds (Ellis et al., 1982). Here artificially created accelerating aging stress causes less effect on transgenic compared to wild-type. The transgenic lines maintained high seedling growth compared to wild-type (**Figures 1C, D**). The marker-free gene containing the construct, *PsAKR1* is possibly involved in nullifying the effect of accelerating aging. This *PsAKR1* gene belongs to the *Aldo ketoreductase* (*AKR*) gene family, which is involved in the detoxification of the reactive carbonyl compounds generated under abiotic stresses (Kirankumar et al., 2016; Nisarga et al., 2017; Nareshkumar et al., 2020). Over-expression of the *PsAKR1* gene in rice and tobacco seedlings showed improved seedling vigor and viability upon accelerating aging (Nisarga et al., 2017).

In addition to salinity stress, the transgenic rice seedlings also showed tolerance to heat stress (**Figures 5A, B**). The study by Qian et al., 2014) showed that overexpression of *HSFA1* in *Arabidopsis* showed enhanced expression of *HSFA1* and also downstream target gene i.e. HSPs under heat and oxidative stress. Heat shock proteins (HSPs), such as HSP90 and HSP70, act as molecular chaperones that facilitate the folding and inhibit their misfoldings under stress conditions (Mohamed and Al-whaibi, 2010; Nakai, 2010). In the present study, the transcript level of HSP was found more in transgenic rice plants compared to wild-type. The result of the study shows that the overexpression of the *HSF4* gene in the transgenic rice could be the contributor to the increased level of HSP transcripts (**Figure 7A**), and it also might be responsible for regulating the increased expression of other downstream stress-responsive genes and protecting the protein functions by chaperon activity in transgenics (**Figure 7C**). Furthermore, methyl viologen-induced oxidative stress caused less effect on transgenic rice plants compared to wild-type (**Figures 4A, B**). The survival of seedlings against oxidative stress depends on the equilibrium between the generation and detoxification of ROS by a series of enzymatic and nonenzymatic mechanisms (Hendadura et al., 2017; Nareshkumar et al., 2020). According to earlier studies, the multi-transgenes used in our study might increase the transcript level of downstream genes, which are involved in switching on the ROS scavenging system under abiotic stress conditions (Kirankumar et al., 2016). Meng et al. (2016) reported that plant *HSFs* are the terminal components of a signal transduction chain mediating the expression of various abiotic stress-responsive genes. Another gene that has been used in our study, i.e. *p68*, showed an increased expression level of ROS scavenging enzymes (*APX* and *SOD*) in various crops like tobacco, rice, and soybean under salt stress (Tuteja et al., 2014; Karthik et al., 2019).

It is well known that whenever moisture stress in rice occurs at the reproductive stage, there will be a considerable reduction in yield since it affects panicle exertion and also induces spikelet sterility (Kamoshita et al., 2004; Ichsan et al., 2020). In our study, transgenic lines showed improved yield compared to wild-type (**Table 1**). It might be due to, transgenic lines having higher productive tillers compared to wild-type. Adaptation to moisture stress under field conditions can be achieved by improving CLT mechanisms besides improving traits associated with water relations. Co-expression of *RNA helicase Pg47*, *Eukaryotic initiation factor 4E* (*eIF4E*) and *Heat shock transcription factor* (*PgHSF4*) in rice showed improved growth and yield by playing a crucial role in removing mRNA secondary structure thereby hastens the translation process and also protect the proteins from degradation under stress condition (Patil thesis, 2015).

Since *Pg47* and *p68* RNA helicases act as functional proteins, which might be interacting with other factors to impart stress tolerance (Tuteja et al., 2014; Karthik et al., 2019). Therefore to find out the interacting proteins, the protein-protein interaction study was carried out with *Pg47* and *p68* genes. The present study found that *Pg47* and *p68* are interacting with other stress-responsive proteins such as *Zinc finger protein*, *eIF4E, eIF4G, eIF4F* (*Eukaryotic translation initiation factors*), *MA3* domain-containing proteins, and *OsRNA helicases* (**Figure S7**). The present study results revealed that the interaction of transgenes and stress-responsive genes-related proteins at the molecular level, all these protein (stress-responsive) interactions might be attributed to stress tolerance by interacting with *p68* and *Pg47*.

## Conclusion

The results of the present study suggest that rice transgenics co-expressing *PgHSF4, p68, Pg47* and *PsAKR1* genes showed significantly improved abiotic stress tolerance in diverse physiological screens. In addition, the study also provided proof of concept that tolerance to semi-irrigated aerobic conditions can be achieved by co-expressing relevant stress-responsive genes in the background of a genotype with superior water relations. Pyramiding cellular level tolerance and water mining traits is a significant approach; contribute to enhanced adaptation and productivity under semi-irrigated aerobic conditions. As a whole, this study provided proof of concept that sustaining the physiological functions, effective management of cytotoxic compounds, and protein stability under stress is a relevant strategy, and these genes can be used as candidate genes for improving abiotic stress tolerance in crop plants. Finally, the multigene transgenics approach could be a potential option to pyramid multiple traits and achieve abiotic stress tolerance.

## Data availability statement

The original contributions presented in the study are included in the article/**Supplementary Material**. Further inquiries can be directed to the corresponding authors.

## Author contributions

PTG and CR designed the experiment, SH conducted the major experiments, and AV assisted in lab experiments. SH and AV wrote the first draft of the manuscript. All authors contributed to the article and approved the submitted version.
