# Supplementary material for: Pyramiding of transcription factor, PgHSF4, and stress-responsive genes of p68, Pg47, and PsAKR1 impart multiple abiotic stress tolerance in rice (Oryza sativa L.)
Source: Front Plant Sci. 2023 Aug 25;14:1233248. doi: 10.3389/fpls.2023.1233248 (PMC10492517; doi:10.3389/fpls.2023.1233248)
Supplement: Supplementary file 1 [file DataSheet_1.docx]

**Table S1: List of the primers used for PCR and semi- quantitative RT –PCR studies of transgenes and downstream genes**

| **Sl. No.** | **Gene Name** | **5’ – 3’ Sequence** | **Annealing Temperature** |
| --- | --- | --- | --- |
| 1 | *PgHSF4 F* | CCTTCGTTGTGGTCAACCGGG | 52 °C |
|  | *PgHSF4 R* | CCTGCGCTCCATATCGATCTTC |  |
| 2 | *P68F* | TCGACGTGGAGATGGTCCCCT | 60 °C |
|  | *P68R* | ACATCCAAGCCCCGTGATGCAA |  |
| 3 | *P68 RTF* | GCGATAGTACGGCTGCACCGG | 58 °C |
|  | *P68 RTR* | GCTCGAAGCCCATATCCAACATGCG |  |
| 4 | *Pg47 F* | AACTTCAGTCCGTCCGTCGAGGACC | 56 °C |
|  | *Pg47 R* | CACGGGATGAACCAGACCTGA |  |
| 5 | *PsAKR1 F* | GATGCACAGAGAGGACGATAGC | 56 ^°^C |
|  | *PsAKR1 R* | ATCAAAGCTGCGCCTTGTAGC |  |
| 6 | *Ubi P F* | GCATATGCAGCAGCTATATGTGG | 58 °C |
|  | *Ubi T R* | CTGCAGAAGTAACACCAAACAAC |  |
| 7 | *RBCS P F* | CCACGTGTCACATCGTCATGGTG | 58 °C |
|  | *RBCS T R* | ATGCGGTTGTAGCATTCC |  |
| 8 | *2x 35S CaMV P F* | AACATGGTGGAGCACGAACA | 58 °C |
|  | *Poly A R P* | ACTGGATTTTGGTTTTAGGAATTAG |  |
| 9 | \| *OsZinc_finger F* \| \| --- \| | GGCATGCTGGATTCAGATTCAG | 56 ^°^C |
|  | \| *OsZinc_finger R* \| \| --- \| | CCTTCCACAGCTCCTGCATC |  |
| 10 | *OsEIF4E F* | CACCGTCGAGGACTTCTGGAG | 58 ^°^C |
|  | *OsEIF4E R* | CTGAGCAGCTTCATTGGCAGC |  |
| 11 | *OsEIF4G F* | CTTGCGACTGTCCTTGGTTC | 56 ^°^C |
|  | *OsEIF4G R* | GATCGACTCTAGAACTGCACC |  |
| 12 | *OsEIF4F* | GCTGGATTCGGTTTTACCAGG | 56 ^°^C |
|  | *OsEIF4R* | CCGCATCCTACTTCACTGATTC |  |
| 13 | *OsMA3_Domain proteinF* | GAGAGGCTTCTGAGATGCTGG | 56 ^°^C |
|  | *OsMA3_Domain protein R* | CAGCAGACCGAGGATCCTGTC |  |
| 14 | *OsRNA_helicase F* | GCTGTATATGAGGCGTGGAAGG | 57 ^°^C |
|  | *OsRNA_helicase R* | GCTGGTTCTCTACCAAGGTTCTG |  |

**Table S2: Seedling growth analysis of T_4_ generation rice transgenics and wild-type upon NaCl-induced stress**

|  | **Seedlings length (cm)** | | | |
| --- | --- | --- | --- | --- |
|  | **Induction treatment** | | **Lethal treatment** | |
|  | **Mean** | **Range** | **Mean** | **Range** |
| **T** | 4.55±0.7 | 3.00-6.29 | 3.22±1.1 | 1.58-4.86 |
| **WT** | 4.14±0.4 | 3.99-4.27 | 2.39±1.5 | 2.01-2.78 |
|  | CD (0.05) = 0.715 | | CD (0.05) = 0.78 | |

(Transgenic lines: nxr = 43x3, WT: nxr =1x10, where, n=number of lines, r=number of replications. T-Transgenics, WT-Wild-type

**Table S3: Seedling growth analysis of T_4_ rice transgenics and wild-type subjected to accelerated aging stress**

|  | **Control** | | | | | **Accelerated aging treatment** | | | | |
| --- | --- | --- | --- | --- | --- | --- | --- | --- | --- | --- |
|  | **Root length** | | **Shoot length** | | | **Root length** | | | **Shoot length** | |
|  | **Mean** | **Range** | **Mean** | | **Range** | **Mean** | **Range** | | **Mean** | **Range** |
| **T** | 3.27±0.59 | 1.74-4.80 | 1.41±0.14 | | 0.78-2.02 | 2.30±0.50 | 1.21-3.36 | | 1.01±0.13 | 0.66-1.28 |
| **WT** | 1.94±0.71 | 1.50-2.45 | 1.00±0.11 | | 0.80-1.20 | 1.17±0.62 | 0.90-1.32 | | 0.91±0.12 | 0.81-1.19 |
|  | CD (0.05) =0.93 | | | CD (0.05) =0.57 | | CD (0.05) =0.72 | | CD (0.05) =0.24 | | |

Transgenic lines: nxr = 43x3, WT: nxr =1x6, where, n=number of lines, r=number of replications. T-, Transgenics WT-Wild-type)

**Table S4: Growth and productivity analysis of T_4_ rice transgenics and wild-type plants under aerobic cultivation (moisture limited stress)**

|  | **TDM(g/plant)** | | **Yield(g/plant)** | | | **No. of productive tillers** | | | **No. of filled seeds** | |
| --- | --- | --- | --- | --- | --- | --- | --- | --- | --- | --- |
|  | **Mean** | **Range** | **Mean** | | **Range** | **Mean** | **Range** | | **Mean** | **Range** |
| **T** | 89.90±9.16 | 70.8-109 | 45.1±8.81 | | 35.2-56.3 | 5.8±1.02 | 5-6.7 | | 1340 | 1250-1420 |
| **WT** | 78.53±1.1 | 75.1-81.9 | 39.9±1.30 | | 37.1-42.8 | 4.5±0.76 | 4-5.0 | | 1115 | 1038-1190 |
|  | CD (0.05) = 26.28 | | | CD (0.05) = 15.39 | | CD (0.05) = 2.704 | | CD (0.05) = 177.9 | | |

(Transgenic lines: nxr = 43x3, WT: nxr =1x6, where, n=number of lines, r=number of replication). T-Transgenics, WT-Wild-type

**A)**

***RBCS*:: *PsAKR1 :: RBCS Ubiq:: p68: :Ubiq 2x 35s::Pg HSF4 ::2x 35s RBCS::Pg47::***

***RBCS***

LB

RB

pi12GW

Spec

**B)**

**Figure S1: Overview of multigene construct:** A) showing the co-expression of *PgHSF4*, *p68,* and *Pg47* genes along with *PsAKR1*, B) Vector map of binary vector (*pi12GW*) backbone expressing the *PsAKR1* as selection marker.

T_0_ – 75 primary transformants were developed

In T_1_ generation, 260 plants were raised based on glyphosate screening

In T_2_ generation, 105 lines were identified and advanced

based on salinity induction response and accelerating aging

From T_2_ generation, 103 lines were advanced to T_3_ generation

Based on salinity induction response, acceleration aging and productivity,

43 lines were identified and advanced to T_4_ generation

Based on productivity, TDM, and molecular characterization,

15 lines were identified and advanced to T_5_ generation.

In T_5_ generation, 15 transgenic lines were characterized based on productivity, molecular analysis, and stress-related assays 1. Seedling level (salinity induction response and TIR) 2. Leaf disc assay

**Figure S2: Workflow chart depicting the generation-wise advancement of rice transgenics.**

**Figure S3: Correlation analysis between drought susceptible index (DSI) and absolute yield value under stress:** Relationship between the DSI and total yield value under stress.


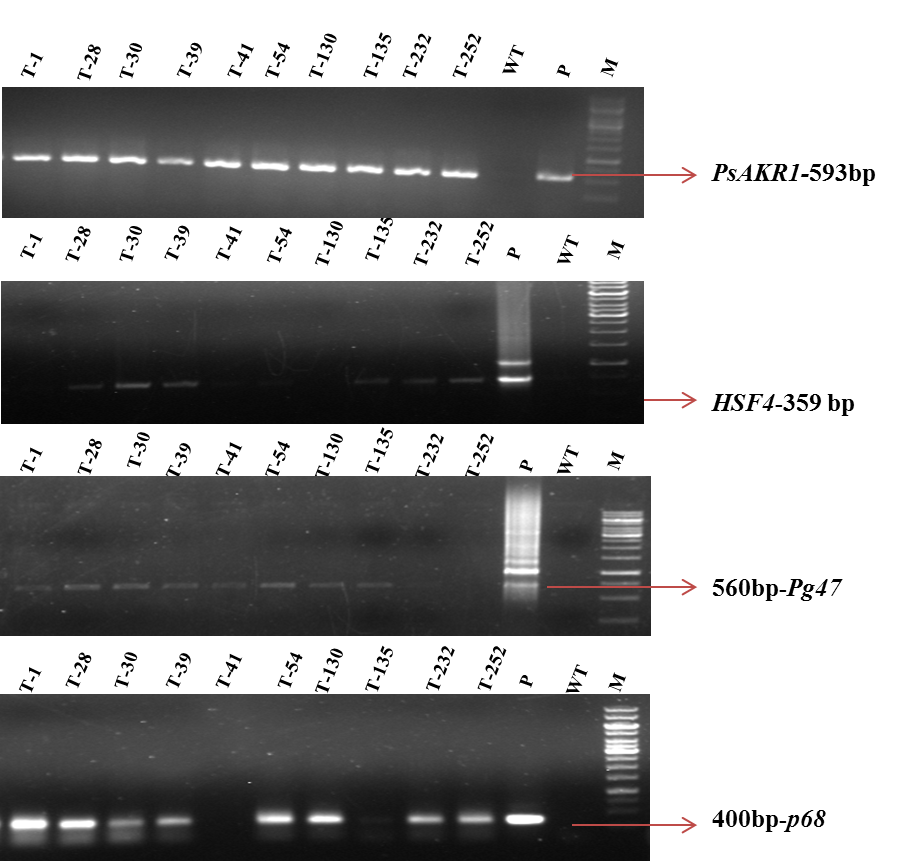


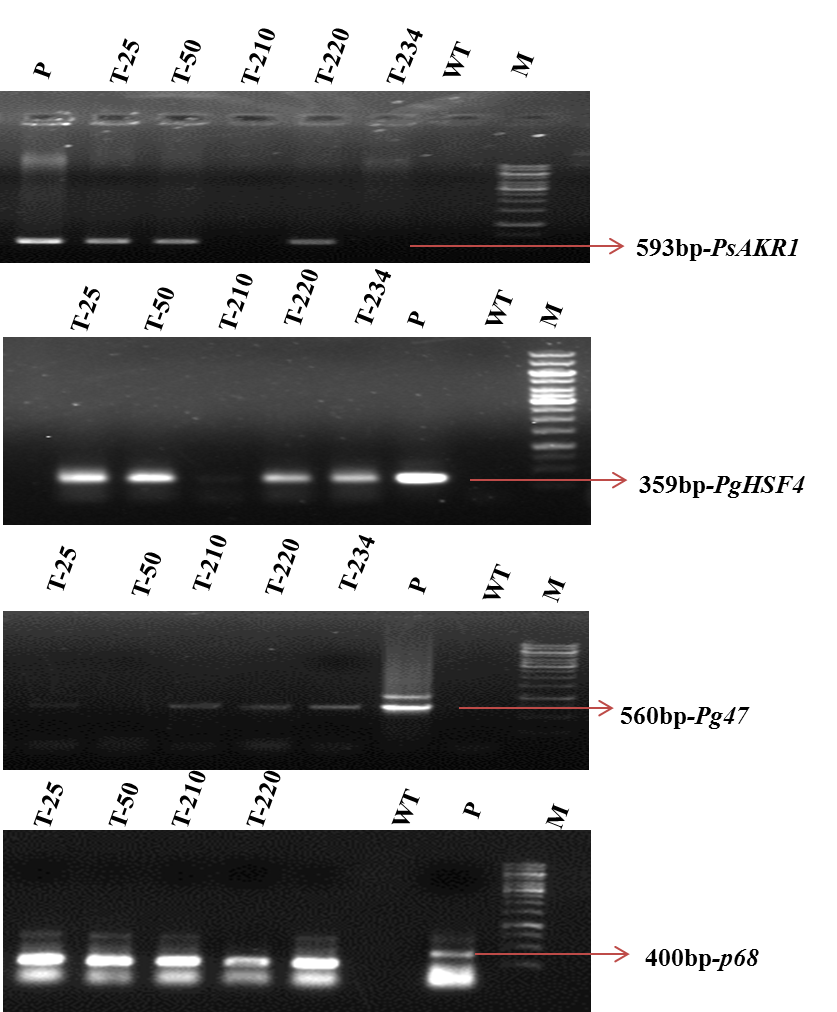


**Figure S4: Molecular characterization of rice transgenic lines**. Genomic DNA was isolated from transgenics and wild-type plants used as a template for PCR amplification. Agarose gel showing PCR amplification of transgenes in rice transgenics. A) PCR showing integration of *PsAKR1* gene, B) *PgHSF4* gene, C) *Pg47*gene and D) *p68* gene (M-1 kb marker, P-plasmid, WT-wild-type, B-Blank, T-Transgenic lines.

**Figure S5: Stable integration of the transgene confirmation by PCR-product gene sequencing.** The PCR-amplified *PsAKR1* gene product was sequenced, and aligned gene sequence analysis was performed using CLASTALW. Sequence 1-Cloned sequence of PsAKR1 gene, 2- PCR product sequence of *PsAKR1* gene.

**A)**


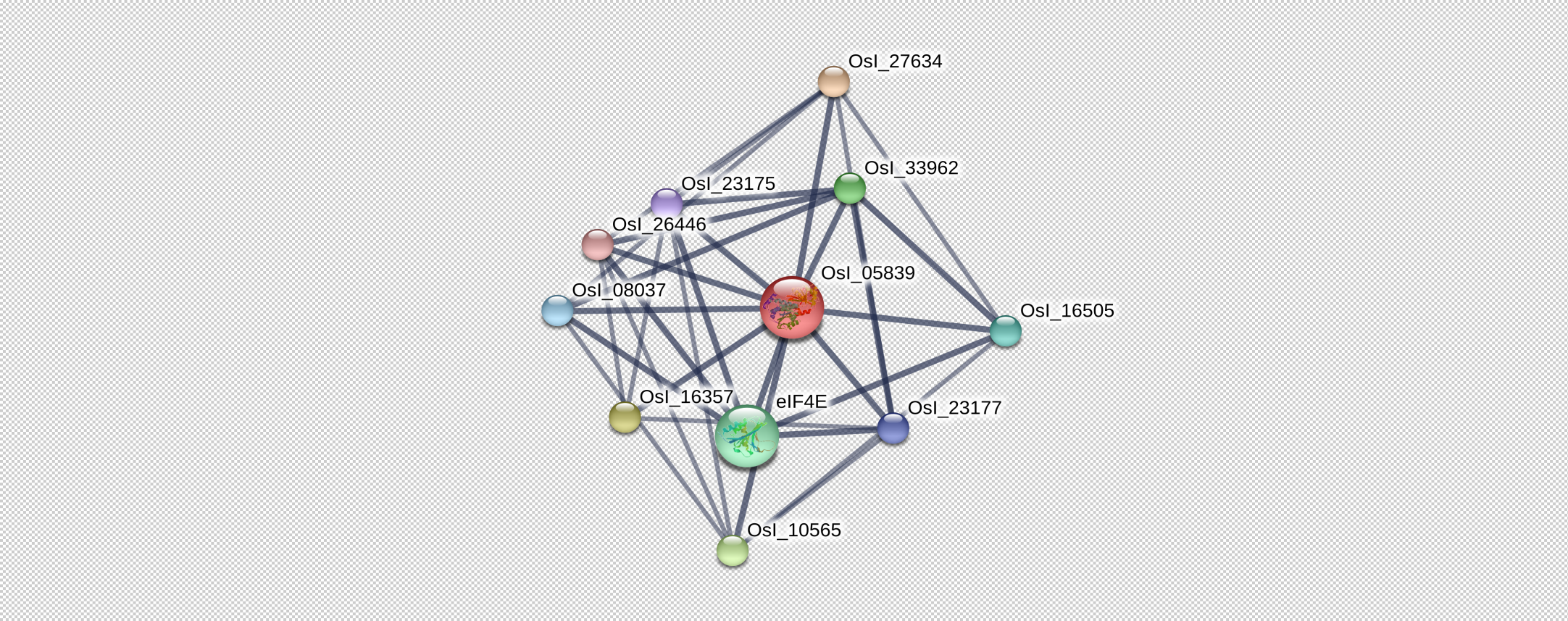


***eIF4E***

**Zinc finger protein**

***MA3 domain containing protein***

***eIF4G***

***Pg47***

***Os RNA helicase***

**B)**


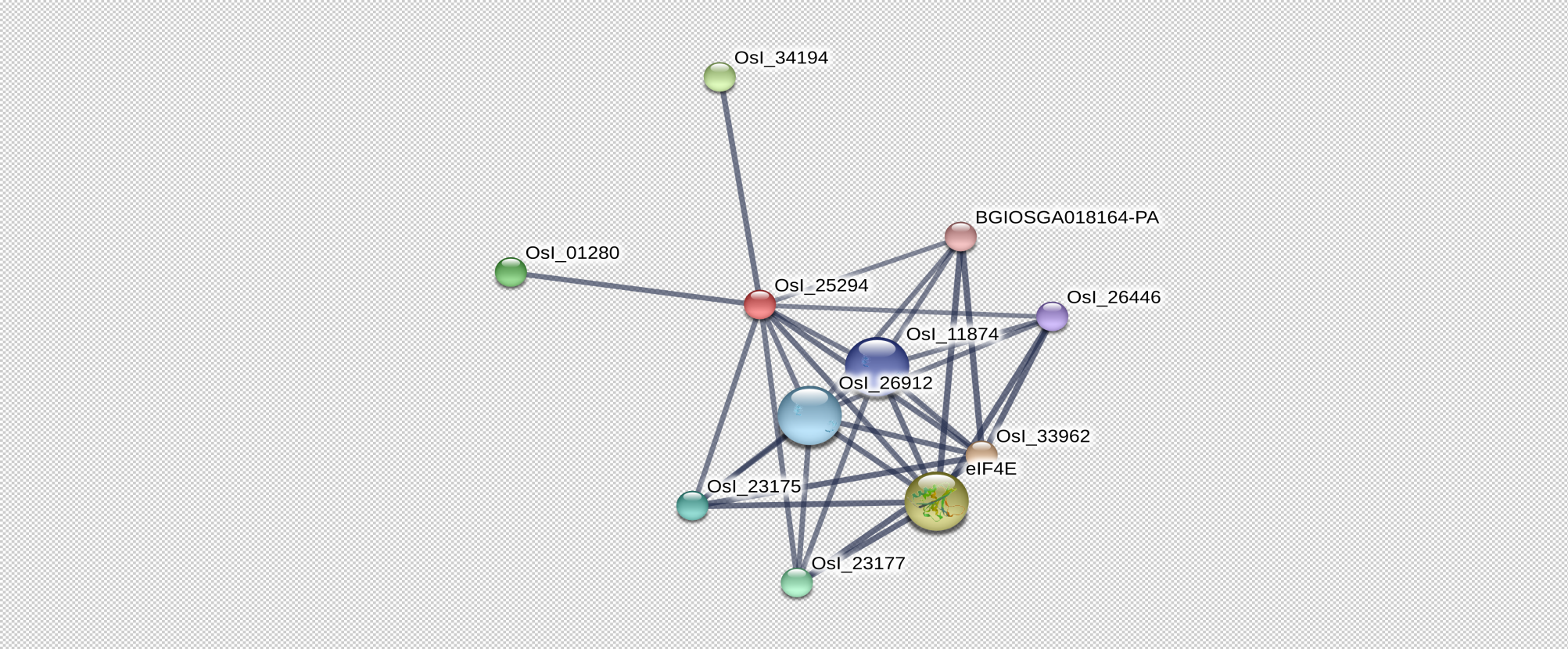


***p68***

***eIF4E***

***eIF4G***

***eIF4F***

***Zinc finger***

***Protein***

***Pre mRNA***

***Processing factor***

**Figure S6: Prediction of interacting proteins for transgenes: A)** Proteins interacting with *Pg47* gene B) Proteins interacting with p68 gene


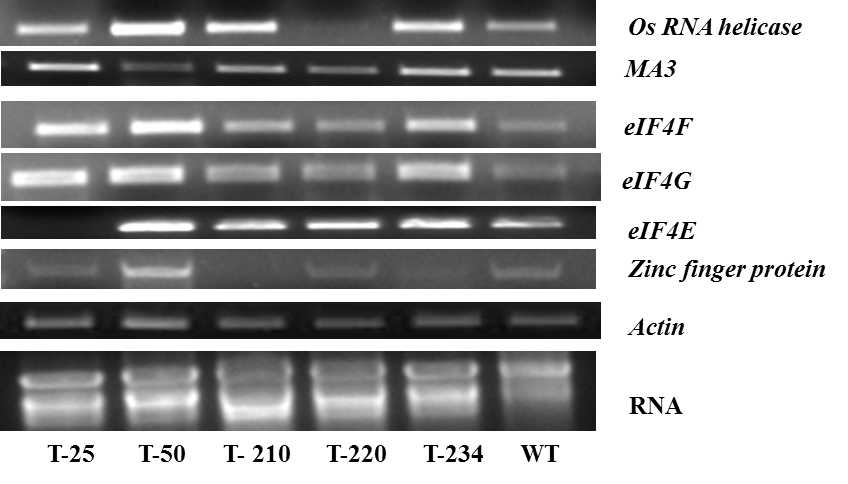


**Figure S7: Expression analysis of downstream targets of genes involved in protein interaction in multigene-expressing transgenic rice plants.** Semi-quantitative gene expression analysis of selected downstream genes in multigene-expressing rice transgenics. Actin was used as a loading control. Total RNA isolated from leaf samples of transgenic and wildtype plants were extracted, and first-strand cDNA was prepared using reverse transcriptase enzyme. It was used as a template for expression analysis. WT – Wild-type, T-25 to T-234 – Transgenic lines
